# P-1342. LiaX Decreases Killing By β-Lactam Antibiotics: Enterococcal Tolerance Revisited

**DOI:** 10.1093/ofid/ofae631.1519

**Published:** 2025-01-29

**Authors:** Ana Streling, Diana Panesso, Kavindra Singh, Sara I Gomez Villegas, Husna Malikzad, William R Miller, Cesar A Arias

**Affiliations:** Houston Methodist Research Institute, Houston, Texas; Houston Methodist Research Institute, Houston, Texas; Houston methodist research institute, Houston, Texas; Brigham and Women's Hospital - Harvard Medical School, Boston, Massachusetts; Houston Methodist Hospital, Houston, Texas; Houston Methodist Research Institute, Houston, Texas; Houston Methodist and Weill Cornell Medical College, Houston, TX

## Abstract

**Background:**

Compared to other Firmicutes, enterococci are often tolerant to many antibiotics; combination therapy is used for severe, deep-seated infections. We have previously reported the LiaFSR system, a three-component cell-envelop stress regulatory system, mediates resistance and tolerance to daptomycin (DAP). The main effector, LiaX, harbors distinct N- and C-terminal domains. The N-terminal portion of LiaX is released into the extracellular milieu where it recognizes DAP and other antimicrobial peptides functioning as a sentinel and signal transduction protein to decrease DAP-mediated killing. LiaX likely acts through LiaF, a transmembrane protein necessary for the LiaX effect. We postulated that the LiaX-LiaF relay system may be involved in the β-lactam tolerance shown by *E. faecalis* (*Efs*).

**Figure 1.**
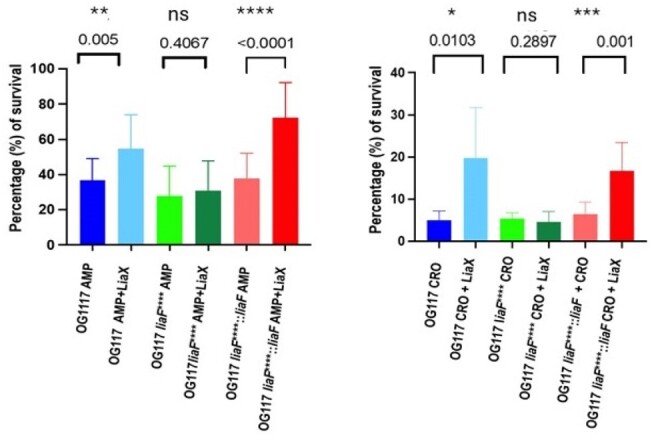
N-terminal domain of LiaX protects E. faecalis from ampicillin and ceftriaxone.

**Methods:**

We used wild type (WT) *Efs* OG117 and derivatives: OG117liaF*_11-14_ (*liaF* deletion) and OG117 liaF*_11-14_::*liaF* (*liaF* complemented). Ampicillin (AMP) and ceftriaxone (CRO) broth-microdilution MICs were performed. N-terminal LiaX_1-275_ was expressed in *E. coli* and purified. Killing assays (5 replicates) with 0.5 X MICs of AMP and CRO were done, with and without LiaX_1-275_ (80 nM). Bacteria were diluted (∼1x10^5^ CFU/mL) and used to inoculate test media containing antibiotic alone, antibiotic+LiaX_1-275_, and no antibiotic. After 2-hours of incubation at 37°C, samples were plated on BHI agar for enumeration.

**Results:**

The CRO MICs for WT and complemented strains were 64 μg/mL, whereas it was 2-fold higher (128 μg/mL) for OG117liaF*_11-14_. All strains showed AMP MICs of 1 μg/mL. In the AMP killing assay, OG117 survival increased with the addition of LiaX_1-275_ (p < .005). The addition of LiaX_1-275_ failed to increase survival of OG117liaF*_11-14_ in the presence of AMP compared to WT (p = 0.4). The survival advantage was restored in the complemented strain (p < .0001). CRO showed a similar trend, i.e., increased survival in OG117 (p < .01) and complemented strain (p < .001), but not in OG117liaF*_11-14_ (p = 0.29).

**Conclusion:**

We have discovered a novel pathway in *Efs* for antibiotic tolerance. The LiaF-LiaX signal relay, part of the enterococcal response to antibiotics, boosts organism survival, contributing to tolerance to multiple anti-enterococcal drugs.

**Disclosures:**

**William R. Miller, M.D.**, Merck: Grant/Research Support|UptoDate: Royalties

